# Development and characterization of type I interferon receptor knockout sheep: A model for viral immunology and reproductive signaling

**DOI:** 10.3389/fgene.2022.986316

**Published:** 2022-09-14

**Authors:** Christopher J. Davies, Zhiqiang Fan, Kira P. Morgado, Ying Liu, Misha Regouski, Qinggang Meng, Aaron J. Thomas, Sang-Im Yun, Byung-Hak Song, Jordan C. Frank, Iuri V. Perisse, Arnaud Van Wettere, Young-Min Lee, Irina A. Polejaeva

**Affiliations:** ^1^ Department of Animal, Dairy and Veterinary Sciences, College of Agriculture and Applied Sciences, Utah State University, Logan, UT, United States; ^2^ Center for Integrated BioSystems, College of Agriculture and Applied Sciences, Utah State University, Logan, UT, United States

**Keywords:** animal models, interferon receptor knockout, type I interferons, innate immunity, Zika virus, recognition of pregnancy, sheep, CRISPR/Cas9

## Abstract

Type I interferons (IFNs) initiate immune responses to viral infections. Their effects are mediated by the type I IFN receptor, IFNAR, comprised of two subunits: IFNAR1 and IFNAR2. One or both chains of the sheep IFNAR were disrupted in fetal fibroblast lines using CRISPR/Cas9 and 12 lambs were produced by somatic cell nuclear transfer (SCNT). Quantitative reverse transcription-polymerase chain reaction for IFN-stimulated gene expression showed that IFNAR deficient sheep fail to respond to IFN-alpha. Furthermore, fibroblast cells from an *IFNAR2* ^***−/−***^ fetus supported significantly higher levels of Zika virus (ZIKV) replication than wild-type fetal fibroblast cells. Although many lambs have died from SCNT related problems or infections, one fertile *IFNAR2* ^***−/−***^ ram lived to over 4 years of age, remained healthy, and produced more than 80 offspring. Interestingly, ZIKV infection studies failed to demonstrate a high level of susceptibility. Presumably, these sheep compensated for a lack of type I IFN signaling using the type II, IFN-gamma and type III, IFN-lambda pathways. These sheep constitute a unique model for studying the pathogenesis of viral infection. Historical data supports the concept that ruminants utilize a novel type I IFN, IFN-tau, for pregnancy recognition. Consequently, IFNAR deficient ewes are likely to be infertile, making IFNAR knockout sheep a valuable model for studying pregnancy recognition. A breeding herd of 32 *IFNAR2* ^***+/−***^ ewes, which are fertile, has been developed for production of *IFNAR2* ^***−/−***^ sheep for both infection and reproduction studies.

## Introduction

The type I interferons (IFNs), IFN-alpha (IFNA, with multiple subtypes) and IFN-beta (IFNB), protect animals against viruses by triggering an innate or early response, which controls viral infections until an adaptive immune response develops (Shaw et al., 2017; Ali et al., 2019; Crow and Ronnblom, 2019). IFNA, IFNB and other type I INFs (IFNW, IFND, IFNE and IFNT) interact with the same IFN receptor, IFNAR, which is a heterodimer comprised of two subunits: IFNAR1 and IFNAR2 (Lim and Langer, 1993; Han et al., 1997; Rosenfeld et al., 2002; Bekisz et al., 2004).

Type I and type II IFN receptor deficient mice constitute important models for studying viral pathogenesis and immunity (Kolokoltsova et al., 2010; Ortego et al., 2014; Lazear et al., 2016). These mice are well characterized small-animal models for studying the neuropathogenesis of Zika virus (ZIKV) and testing the efficacy of antivirals and vaccines. The mouse models provide several advantages: 1) mice are small and relatively easy to handle at a high biosafety level; 2) the murine central nervous system (CNS) is extensively studied and relatively well characterized, allowing extrapolation of findings to the human CNS; 3) there are plenty of available reagents for neuropathogenetic studies; and 4) mouse genetics are readily available for genome manipulation (e.g., CRISPR/Cas9 technology). There are, however, significant differences in the innate and adaptive immune systems of mice and humans and, therefore, they often respond differently to pathogens (Mestas and Hughes, 2004; Seok et al., 2013). Furthermore, in the context of pregnancy and fetal brain development there are disadvantages of using mice to study ZIKV infection. Mice have a much shorter gestation period than humans, only about 20 days, and the structure of their placenta and brain differs significantly (Coyne and Lazear, 2016), making it difficult to recapitulate congenital Zika syndrome. In contrast, sheep have a long gestation period of about 5 months, and their brain has similar anatomy and physiology to the human brain, although their placenta differs. A type I interferon receptor (IFNAR) knockout sheep model would thus provide a better animal model, or a good alternative model, for studying the pathogenesis of ZIKV and other closely related flaviviruses, particularly during pregnancy. To meet the need for a better large animal model for studying infectious diseases, we developed the genetically engineered, IFNAR knockout sheep model described here.

Ruminants are unique in that they utilize a type I IFN, IFN-tau (IFNT), for pregnancy recognition (Roberts et al., 2016; Yoshinaga, 2018). IFNT appears to have evolved from IFNW and, at least in cattle, is encoded by three duplicated genes (Winkelman et al., 1999; Walker et al., 2009; Ealy and Wooldridge, 2017; Ezashi and Imakawa, 2017). At a critical time in early gestation (days 12–15 in sheep and goats), trophoblast cells of the placenta secrete IFNT, which signals the mother to maintain her corpus luteum (CL) and not return to estrus. IFNAR is expressed on luminal and superficial glandular endometrial epithelial cells that are the primary target for IFNT (Imakawa et al., 2002; Rosenfeld et al., 2002). In response to IFNT, endometrial epithelial cells down-regulate expression of estrogen and oxytocin receptors, (Mirando et al., 1990; Vallet and Lamming, 1991; Ott et al., 1992; Lamming et al., 1995; Spencer and Bazer, 1996; Spencer et al., 1996; Bazer et al., 1997; Spencer et al., 1998; Hansen et al., 2017; Bazer et al., 2018), making them refractory to the pulsatile release of oxytocin from the ovary and abolishing their pulsatile release of prostaglandin F2 alpha (PGF2α; McCracken et al., 1984; Hansen et al., 2017). During the estrus cycle, PGF2α is carried directly to the ovary by a countercurrent exchange mechanism (McCracken et al., 1984). In the ovary, the pulses of PGF2α trigger regression of the CL and initiation of estrus (McCracken et al., 1984; Hansen et al., 2017). In early pregnancy, activation of IFNAR by IFNT blocks the release of PGF2α and subsequent regression of the CL, allowing development of the conceptus to continue and pregnancy to become established (Bazer et al., 1997; Hansen et al., 2017). IFNT is believed to work exclusively through IFNAR.

In this report, we describe our initial experiments addressing two hypotheses. The first hypothesis is that *IFNAR2* ^***−/−***^ sheep will be more susceptible to viral infections and will respond differently to viral infections than normal sheep. The second hypothesis is that *IFNAR2* ^***−/−***^ ewes will be sterile due to an inability to respond to IFNT, the pregnancy recognition factor in ruminants.

## Materials and methods

### Somatic cell nuclear transfer recipients

Domestic sheep (*Ovis aries*) were used as somatic cell nuclear transfer (SCNT) recipients. Sheep were housed at the Utah State University, Animal Science Farm. Forty-eight parous ewes between two and 5 years of age, of various breeds were used as embryo recipients. All animal procedures were approved by the Utah State University, Institutional Animal Care and Use Committee (Protocol numbers: 10127, 10198 and 11498).

### Zika virus strains

We used three genetically distinct strains of ZIKV: 1) MR-766, which was isolated in 1947 from a *Macaca mulatta* monkey in Uganda and represents an African lineage; 2) P6-740, which was isolated in 1966 from a pool of *Aedes aegypti* mosquitoes in Malaysia and represents an Asian lineage; and 3) PRVABC-59, which was isolated in 2015 from a human patient in Puerto Rico and is an Asian lineage-derived American strain (Yun et al., 2016a). For all three ZIKV strains, viral stocks were generated from their full-length infectious cDNA molecular clones in Vero cells, and their virological properties were previously characterized, both in cell cultures and in mice (Yun et al., 2018).

### Production of knockout cell lines

Primary sheep fetal fibroblasts (SFFs) were isolated from day 45 Romney fetuses and cultured in high-glucose Dulbecco’s Modified Eagle Medium (HyClone, Logan, UT, United States) supplemented with 15% fetal bovine serum (FBS; HyClone, Logan, UT, United States) and 100 U/ml penicillin/streptomycin (Life Technologies, Carlsbad, CA, United States) at 38.5°C in an atmosphere of 5% CO_2_ in air (Fan et al., 2018). The sex of SFF cell lines was determined by PCR amplification of the SRY gene located on the Y chromosome (Saberivand and Ahsan, 2016). CRISPR/Cas9 gene-targeting vectors were constructed using the pX330-U6-Chimeric_BB-CBh-hSpCas9 plasmid (pX330; Addgene, Watertown, MA, United States) as described by Cong et al. (Cong et al., 2013). The sgRNA/Cas9 target sites for each exon of interest were identified by searching for G(N)_20_GG motifs. The corresponding oligonucleotides for each target site were purchased from Integrated DNA Technologies (Coralville, IA, United States). The final constructs were confirmed by Sanger sequencing, performed by the Utah State University, Center for Integrated BioSystems, Genomics Core Laboratory (Logan, UT, United States) using an ABI PRISM 3730 DNA Analyzer (Applied Biosystems, Bedford, MA, United States). Five micrograms of circular sgRNA/Cas9 vectors were transfected into SFFs at early passages using an Amaxa 4D-Nucleofector (program no. EH-100; Lonza, Morristown, NJ, United States). Three days after transfection, cells were harvested for genomic DNA isolation by using a DNeasy Blood & Tissue Kit (QIAGEN, Germantown, MD, United States) following the manufacturer’s protocol. Each targeted genomic locus was PCR-amplified from SFF genomic DNA by Phusion High-Fidelity DNA Polymerase (Thermo Fisher Scientific, Waltham, MA, United States). After digestion with an appropriate restriction enzyme, PCR products were resolved on a 1% agarose gel and stained with SYBR green dye (Life Technologies, Carlsbad, CA, United States). Indels were detected based on whether the PCR products were fully or partially resistant to digestion by a restriction enzyme that cuts at the target site, i.e., PCR-restriction fragment length polymorphism (PCR-RFLP) assays; indels introduced by sgRNA/Cas9 vectors abolish the restriction recognition sites. To determine gene targeting efficiency by each of the sgRNA/Cas9 vectors, the relative intensities of the uncut and cut bands were analyzed using NIH ImageJ software 1.47p. Three days after targeting vector transfection, cells were subjected to single-cell cloning by serial dilution in 96-well plates. Each single-cell colony was allowed to grow to near confluence. PCR-RFLP assays and Sanger sequencing were used for identification of *IFNAR1* ^***−/−***^ or *IFNAR2* ^***−/−***^ mutated colonies.

### Somatic cell nuclear transfer

Sheep SCNT was performed as described by Yang et al. for goats (Yang et al., 2016) with minor modifications wherein an aspiration technique was used for oocyte recovery instead of a slicing technique. *IFNAR1* ^***−/−***^ and *IFNAR2* ^***−/−***^ fetal fibroblasts were grown to 80%–90% confluence and used as nuclear donors for SCNT after 24 h of serum starvation (0.5% FBS). The cloned embryos were cultured in Synthetic Oviductal Fluid medium for 10–12 h and then transferred into estrus-synchronized recipients.

### Genotyping of lambs produced by SCNT and breeding

Initially genotyping of lambs was done using the same PCR-RFLP assays used for characterization of the *IFNAR1* and *IFNAR2* knockout cell lines. In addition, Sanger sequencing was used to confirm the sequence of the indels. Surprisingly, breeding of one of the three *IFNAR2* ^***−/−***^ rams produced by SCNT from colony #57 revealed that approximately half of his offspring did not have a detectable knockout allele. Because this ram lacked a wild-type (WT) allele and only had one known knockout allele with a 5 bp deletion, we concluded that he had to have a large deletion on one chromosome. Consequently, we designed several PCR primers further away from the target site and eventually succeeded in amplifying an allele with a large 1,282 bp deletion (for primer sequences see Table 1). The new primer set was used with the restriction enzyme NcoI to detect the WT and both knockout alleles in this line of sheep.

**TABLE 1:** PCR primers for the amplification of *IFNAR1* and *IFNAR2*.

Gene/Exon	Sequence (5′–3′)	Amplicon (bp)
*IFNAR1*/Exon 2	Forward: CTT​CCC​ATG​CTG​AGA​ACA; Reverse: GTCGGAACTCTTAGACCA	491
*IFNAR1*/Exon 4	Forward: AGT​CGT​TGC​CAC​CCT​TCT; Reverse: TCG​GGA​TAA​ACA​GTT​TCA​GT	529
*IFNAR2*/Exon 1	Forward: AGC​GTT​TCT​CGT​GAT​GTA; Reverse: TCCACTTGGCTCTTGACC	418
*IFNAR2*/Exon 1^a^	Forward: CCA​TTC​TTT​ACA​CTG​AGG​ATA​C; Reverse: GCT​ATT​GGT​CCT​GCA​AGC​T	1,706
*IFNAR2*/Exon 3	Forward: TGG​TGC​TGA​GTT​CCT​GTA; Reverse: TGTAGAAATTGGCTTTGG	593
*IFNAR2*/Exon 4	Forward: ACC​TTT​GAG​CAG​AGC​CAC​A; Reverse: AGAAGCCAAGTAACCACT	442

a This primer set was created to detect a large 1,282 bp deletion found in the *IFNAR2* ^***−/−***^ rams from colony #57.

### Off-target analysis

The Benchling CRISPRtool (https://www.benchling.com/) was used to search the sheep genome database for genomic sequences with the highest homology to the *IFNAR1* and *IFNAR2* target sequences. Ten potential off-target loci with >0.5% probability of being targeted were selected for each gene. Specific PCR primers were designed to amplify DNA fragments from 320 to 740 bp in length spanning each off-target locus (Supplemental Table S1). PCR amplification and Sanger sequencing for off-target analysis were performed with genomic DNA isolated from a single *IFNAR1* ^***−/−***^ and four *IFNAR2* ^***−/−***^ sheep.

### Assessment of cellular response to IFNA

Fibroblast cultures were established using skin biopsies from knockout and WT sheep. Fibroblasts (2.0 × 10^5^) were cultured in complete media for 24 h; then recombinant human IFNA (100 ng/ml; Novus Biologicals, Centennial, CO, United States) was added to one of two replicate cultures and the cells were cultured for an additional 24 h. Expression of IFN stimulated genes (ISGs) was analyzed by quantitative reverse transcription-polymerase chain reaction (qRT-PCR) using a Biomark microfluidic qPCR system (Fluidigm, South San Francisco, CA, United States). Primers for four housekeeping genes (*GAPDH*, *ACTB*, *YWHAZ*, and *EIF4A1*) and five ISGs (*MX1*, *MX2*, *IRF2*, *ISG15*, and *B2M*) were used (Supplemental Table S2). The *IFNAR1* ^***−/−***^ and *IFNAR2* ^***−/−***^ cultures were treated as a single group because both failed to respond to IFNA. Fold changes between unstimulated and stimulated cultures were calculated by the ΔΔCt method using the average of the four housekeeping genes for normalization (Livak and Schmittgen, 2001).

### Zika virus replication in fetal fibroblasts

One day prior to infection, primary fibroblasts isolated from WT and *IFNAR2* ^*−/−*^ fetuses were seeded into 35 mm culture dishes at a density of 3 × 10^5^ cells/dish. Both WT and *IFNAR2* ^*−/−*^ cell monolayers were infected with each of three different strains of ZIKV (MR-766, P6-740, and PRVABC-59) at a multiplicity of infection of one for 1 h at 37°C. The infected cells were then washed once with complete culture medium and incubated for 4 days. During the incubation, culture supernatants were collected at 6, 12, 18, 24, 36, 48, 60, 72, and 96 h after infection and used for quantification of virus production by a standard plaque assay on Vero cells as described previously (Yun et al., 2016b; Yun et al., 2018). In brief, Vero cells were seeded in a 6-well plate at a density of 3 × 10^5^ cells/well for 12 h, infected in duplicates with serial 10-fold dilutions of culture supernatants in a final volume of 1 ml for 1 h at 37°C, overlaid with 3 ml of α-minimal essential medium containing 0.5% agarose and 10% FBS, and then incubated for 4 days. Viral plaques were visualized by fixation with 7% formaldehyde and counterstaining with 1% crystal violet.

### Zika virus replication in animals

All sheep infection experiments were carried out in strict accordance with the recommendations in the Guide for the Care and Use of Laboratory Animals of the National Institutes of Health. The animal protocol was approved by the Institutional Animal Care and Use Committee of Utah State University (Protocol number: 10198). For infection experiments, groups of two, 1-month-old WT and *IFNAR2* ^*−/−*^ ewe lambs were inoculated intravenously with 2.0 × 10^6^ PFU/animal of ZIKV PRVABC-59 in 1 ml of α-minimal essential medium and monitored daily for any clinical signs and weight changes for 19 days. Also, blood samples were taken daily for the quantification of viral genomic RNA by qRT-PCR, as described previously (Yun et al., 2018). In brief, viral genomic RNA was extracted from equal volumes of serum using Trizol LS reagent (Invitrogen, Waltham, MA, United States) as recommended by the manufacturer. The purified viral RNA was used to synthesize its cDNAs with Superscript III reverse transcriptase (Invitrogen, Waltham, MA, United States). The cDNAs were amplified with the iQ Supermix reagent (Bio-Rad, Hercules, CA, United States) and detected with the 7500 Fast Real-Time PCR system (Applied Biosystems, Bedford, MA, United States) using forward and reverse primers (GAA​GTG​GAA​GTC​CCA​GAG​AG and TGC​TGA​GCT​GTA​TGA​CCC​G) and a fluorogenic probe (*FAM*-TGGAGCTCAGGCTTTGATTGGGTGAC-*BHQ*) specific for the ZIKV NS3 protein-coding region. The quantity of viral genomic RNA was determined based on a standard curve generated using a full-length infectious cDNA clone of ZIKV PRVABC-59 (Yun et al., 2018).

### Development of an *IFNAR2* ^*+/−*^ breeding herd

Producing animals by SCNT is very inefficient, and female *IFNAR1* ^**−/−**^ or *IFNAR2* ^**−/−**^ sheep are likely infertile. Consequently, we elected to produce a breeding herd of *IFNAR2* ^**+/−**^ ewes. This breeding herd was produced by breeding an *IFNAR2* ^**−/−**^ Romney ram produced by SCNT to 25 WT Romney ewes in the fall of 2018 and 2019. Subsequently, *IFNAR2* ^**+/−**^ ewes were bred to *IFNAR2* ^**−/−**^ or *IFNAR2* ^**+/−**^ rams for production of replacement breeding stock and *IFNAR2* ^***−/−***^ sheep for experimental studies.

## Results

### Production of knockout cell lines

**FIGURE 1:**
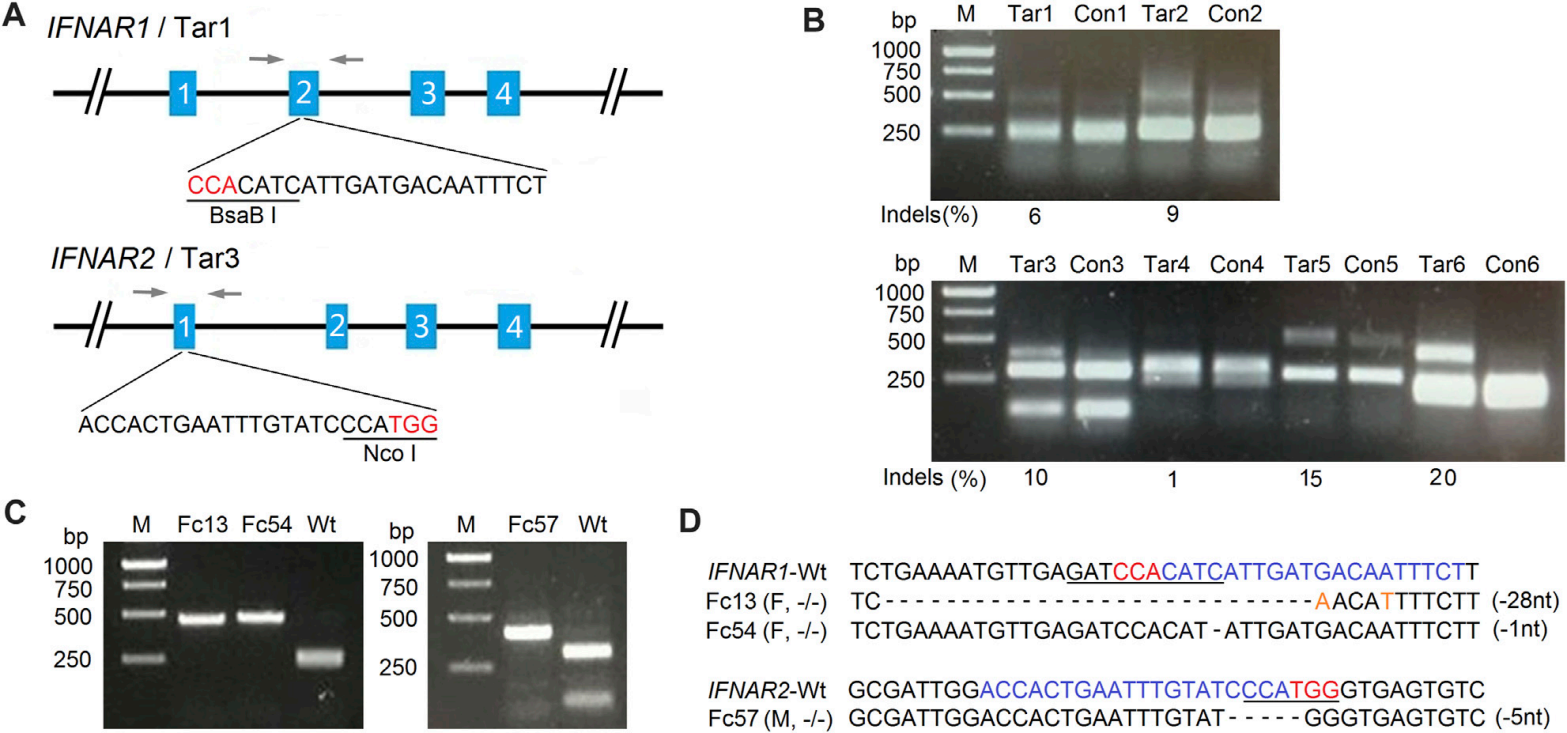
Generation of *IFNAR1* ^**−/−**^ and *IFNAR2* ^**−/−**^ sheep fetal fibroblast colonies for SCNT by CRISPR/Cas9. **(A)** Schematic diagram of *IFNAR1* and *IFNAR2* targeting sites. The single-guide RNA (sgRNA) target sequences for each locus are depicted, with the restriction enzyme recognition sites used for PCR-RFLP assays underlined. Letters in red indicate protospacer-adjacent motifs (PAMs). Arrows indicate locations of PCR primers. **(B)** Gene targeting efficiency analysis of two targeting vectors for the *IFNAR1* locus (upper panel) and four targeting vectors for the *IFNAR2* locus (lower panel) in SFFs detected by PCR-RFLP assays. M, 1-kb DNA ladder; Con, control (WT SFFs); Tar, SFFs transfected with each targeting vector. The targeted alleles lost restriction sites through error-prone non-homologous end joining (NHEJ) following Cas9-mediated double-stranded DNA breaks. The mutation efficiency (indels) of targeting vector 1 (Tar1) was 6% and of targeting vector 3 (Tar3) was 10%. These are the targeting vectors that were used for the generation of the *IFNAR1* ^**−/−**^ and *IFNAR2* ^**−/−**^ sheep fetal fibroblast colonies, respectively. **(C)** PCR-RFLP assays for the detection of *IFNAR1* ^**−/−**^ and *IFNAR2* ^**−/−**^ single-cell derived SFF colonies with mutations at *IFNAR1* (left) and *IFNAR2* (right). **(D)** Sequencing analysis of *IFNAR1* ^**−/−**^ and *IFNAR2* ^**−/−**^ colonies. Letters in blue indicate sgRNA sequences and orange nucleotides replaced at cleavage sites. M, male; F, female; −/−, both alleles targeted; –1 nt, one nucleotide deletion.

Primary fetal fibroblasts from domestic sheep (*Ovis aries*) were used since they are the cells of choice for transgenic SCNT (Schnieke et al., 1997; Polejaeva et al., 2016; Fan et al., 2018). Specific PCR primers were designed based on the sheep *IFNAR1* and *IFNAR2* genome sequences (GenBank, NC_019458.2) and used to amplify corresponding exons and partial intron sequences (Table 1). The sgRNAs were designed targeting different exons of *IFNAR1* and *IFNAR2*, with specific restriction enzyme sites present at the targeted location to facilitate mutation detection (Table 2; Figures 1A,B). A pair of oligonucleotides for each targeting site was synthesized and ligated to the pX330 vector (Table 2) as previously described (Cong et al., 2013). Three days after SFFs were transfected with targeting vectors, gene mutation efficiencies were determined by PCR-RFLP assays. The mutation efficiency of targeting vectors ranged from 6%–9% for *IFNAR1* and 1%–20% for *IFNAR2* (Figure 1B). Single cell derived, mutated fibroblast colonies were isolated by limiting dilution and screened by PCR-RFLP assays (Figure 1C). Targeted biallelic disruption was achieved both in female colonies for *IFNAR1* and male colonies for *IFNAR2*, with screening efficiencies of 12.0 and 2.2%, respectively (Table 3). Sequence analysis of the PCR products indicated that mutations included nucleotide replacements and both small and large indels introduced at the loci targeted in these colonies (Figure 1D). Furthermore, after co-transfection with vectors targeting *IFNAR1* and *IFNAR2*, both male and female, double gene mutated colonies were obtained (Supplemental Table S3).

**TABLE 2:** DNA oligonucleotides for the construction of CRISPR/Cas9 targeting vectors.

Targeting vector	Gene/Exon	Enzyme	Name	Sequence (5′–3′)
Tar1	*IFNAR1*/Exon 2	BsaBI	Cri-sgRNA-1F	CAC​CGA​GAA​ATT​GTC​ATC​AAT​GAT​G
			Cri-sgRNA-1R	AAA​CCA​TCA​TTG​ATG​ACA​ATT​TCT​C
Tar2	*IFNAR1*/Exon 4	BmgBI	Cri-sgRNA-2F	CAC​CGC​TTC​TAA​ATG​CAC​GTC​TGG
			Cri-sgRNA-2R	AAA​CCC​AGA​CGT​GCA​TTT​AGA​AGC
Tar3	*IFNAR2*/Exon 1	NcoI	Cri-sgRNA-3F	CAC​CGA​CCA​CTG​AAT​TTG​TAT​CCC​A
			Cri-sgRNA-3R	AAA​CTG​GGA​TAC​AAA​TTC​AGT​GGT​C
Tar4	*IFNAR2*/Exon 3	ApoI	Cri-sgRNA-4F	CAC​CGA​TAA​GAT​TGA​CTG​GAA​ATT​T
			Cri-sgRNA-4R	AAA​CAA​ATT​TCC​AGT​CAA​TCT​TAT​C
Tar5	*IFNAR2*/Exon 3	MunI	Cri-sgRNA-5F	CAC​CGA​GTG​AGT​TGG​TAC​AAT​TGA​A
			Cri-sgRNA-5R	AAA​CTT​CAA​TTG​TAC​CAA​CTC​ACT​C
Tar6	*IFNAR2*/Exon 4	BtsI	Cri-sgRNA-6F	CAC​CGA​TAC​AGA​GAG​AAC​GCA​GTG​G
			Cri-sgRNA-6R	AAA​CCC​ACT​GCG​TTC​TCT​CTG​TAT​C

**TABLE 3:** Characterization of fibroblast colonies with mutations in *IFNAR1* or *IFNAR2*.

Cell line (sex)	Gene/Exon/Targeting vector	No. of colonies isolated	No. of colonies with mutations (%)	Monoallelic disruption (%)	Biallelic disruption (%)
SFF3 (F)	*IFNAR1*/Exon 2/Tar1	83	13 (15.7)	3 (3.6)	10 (12.0)
SFF5 (M)	*IFNAR2*/Exon 1/Tar3	89	6 (6.7)	4 (4.5)	2 (2.2)

### Somatic cell nuclear transfer

The results for the transfer of cloned embryos are summarized in Table 4. In 2016, embryos were produced from one male *IFNAR2* ^***−/−***^ and two female *IFNAR1* ^***−/−***^ colonies by SCNT; 130 one-cell stage embryos were surgically transferred into nine estrus synchronized recipient sheep. One male fetus was collected at 1 month of gestation for isolation of fibroblast cells. Three male and two female lambs were born in 2017 but one female lamb died shortly after birth. In 2017, 222 embryos from four female *IFNAR2* ^**−/−**^ cell lines were transferred into 13 recipients. Three lambs were born alive but died from polioencephalomalacia, which was likely caused by thiamine deficiency, at about 2.5 months of age. In 2018, *IFNAR1* and *IFNAR2* were knocked out simultaneously in male and female cell lines. A total of 408 embryos were transferred into 26 recipients. No lambs with biallelic knockout of both genes were produced. However, three ewe lambs with an *IFNAR1* ^**+/−**^, *IFNAR2* ^**−/−**^ genotype were born alive. One lamb died from sever hydronephrosis resulting in kidney failure, which is a complication frequently associate with SCNT (Wells et al., 1997), at 1 day of age. The other two lambs were used for a ZIKV infection experiment (see below).

**TABLE 4:** Development rates following SCNT for single and double gene knockout colonies.

Year	KO colony # (sex)	Cell line	Genotype & exon targeted	No. of embryos transferred/No. of recipients	Pregnancy rate (%)	Term rate (%)	No. Alive at 1 month of age
2016	#13 (F)	SFF3	*IFNAR1* ^**−/−**^, Exon 2	44/3	1/3 (33.3)	1/3 (33.3)	0
	#54 (F)	SFF3	*IFNAR1* ^**−/−**^, Exon 2	32/2	1/2 (50.0)	1/2 (50.0)	1
	**Total *IFNAR1*** ^**−/−**^	**SFF3**	***IFNAR1* ^−/−^, Exon 2**	**76/5**	**2/5 (40.0)**	**2/5 (40.0)**	**1**
	#57 (M)	SFF5	*IFNAR2* ^**−/−**^, Exon 1	54/4	4/4 (100)	3/3 (100) ^a^	3
	**Total *IFNAR2*** ^**−/−**^	**SFF5**	***IFNAR2* ^**−/−**^, Exon 1**	**54/4**	**4/4 (100)**	**3/3 (100)** **^a^**	**3**
2017	#A3 (F)	SFF3	*IFNAR2* ^**−/−**^, Exon 1	54/3	3/3 (100)	1/3 (33.3)	0
	#A64 (F)	SFF3	*IFNAR2* ^**−/−**^, Exon 1	36/2	0/2 (0)	0	0
	#A89 (F)	SFF3	*IFNAR2* ^**−/−**^, Exon 1	60/3	3/3 (100)	3/3 (100)	3
	#B20 (F)	SFF3	*IFNAR2* ^**−/−**^, Exon 1	72/5	3/5 (60.0)	0/3 (0)	0
	**Total *IFNAR2*** ^**−/−**^	**SFF3**	***IFNAR2* ^**−/−**^, Exon 1**	**222/13**	**9/13 (69.2)**	**4/9 (44.4)**	**3** **^b^**
2018	#A6 (F)	SFF3	*IFNAR1* ^**−/−**^, Exon 2	31/2	1/2 (50.0)	1/1 (100)	0
			*IFNAR2* ^**−/−**^, Exon 1				
	#A25 (F)	SFF3	*IFNAR1* ^**−/−**^, Exon 2	40/3	0/3 (0)	0	0
			*IFNAR2* ^**−/−**^, Exon 1				
	#C54 (F)	SFF3	*IFNAR1* ^**−/−**^, Exon 2	38/3	0/3 (0)	0	0
			*IFNAR2* ^**−/−**^, Exon 1				
	**Total female double gene KO**	**SFF3**	***IFNAR1* ^**−/−**^, Exon 2**	**109/8**	**1/8 (12.5)**	**1/1 (100)**	**0**
			***IFNAR2* ^−/−^, Exon 1**				
	#A56 (M)	SFF5	*IFNAR1* ^**−/−**^, Exon 2	81/4	1/4 (25.0)	0/1 (0)	0
			*IFNAR2* ^**−/−**^, Exon 1				
	#B29 (M)	SFF5	*IFNAR1* ^**−/−**^, Exon 2	47/3	0/3	0	0
			*IFNAR2* ^**−/−**^, Exon 1				
	#B64 (M)	SFF5	*IFNAR1* ^−/−^, Exon 2	49/4	0/4	0	0
			*IFNAR2* ^**−/−**^, Exon 1				
	**Total male double gene KO**	**SFF5**	***IFNAR1* ^**−/−**^, Exon 2**	**177/11**	**1/11 (9.1)**	**0/1 (0)**	**0**
			***IFNAR2* ^−/−^, Exon 1**				
	#A46 (F) ^c^	SFF3	*IFNAR1* ^***+/−***^, Exon 2	122/7	4/7 (57.1)	2/4 (50.0)	2
			*IFNAR2* ^**−/−**^, Exon 1				
	**Total female IFNAR1^+/−^, IFNAR2^−/−^**	**SFF3**	***IFNAR1* ^***+/−***^, Exon 2**	**122/7**	**4/7 (57.1)**	**2/4 (50.0)**	**2**	
			***IFNAR2* ^−/−^, Exon 1**					

a One pregnancy was terminated at 1 month of gestation for isolation of *IFNAR2* ^**−/−**^ fibroblast cells.

b Three lambs were born alive but died from polioencephalomalacia at about 2.5 months of age.

c It was determined that this colony was only a monoallelic knockout for *IFNAR1* after the lambs were born.

Group totals are in bold text.

### Evaluation of potential mutations at off-target sites by CRISPR/Cas9

To investigate whether unexpected off-target events occurred during genome editing, the sheep genome sequence database was screened using the *IFNAR1* and *IFNAR2* target sequences to identify regions with the highest homologies. In total, 20 potential off-target sites were selected for analysis by PCR amplification and Sanger sequencing (Table 5 and Supplemental Table S1). Analysis was conducted using genomic DNA isolated from a single *IFNAR1* ^***−/−***^ (7IFN3) and four *IFNAR2* ^***−/−***^ (7IFN1, 7IFN2, 7IFN4, and 7IFN-fetus) sheep. None of the *IFNAR1* ^***−/−***^ or *IFNAR2* ^***−/−***^ sheep had indels at the analyzed off-target sites (Supplemental Figure S1). The only potential mutation was at the *IFNAR1* OFT10 site. However, although the OFT10 site contained four nucleotides that differed from the database sequence, comparison with a sequence from a WT Romney sheep confirmed that this sequence was normal for Romney sheep, indicating that there was not an off-target mutation produced by CRISPR/Cas9.

**TABLE 5:** Potential off-target sites for *IFNAR1* and *IFNAR2* targeting vectors.

On/off target		Locus	Chromosome	Sequence	Mismatch	Score
On target		IFNAR1	chr1	AGA​AAT​TGT​CAT​CAA​TGA​TG TGG	-	-
Off target	OFT1	5,008	chr8	TGA​AGT​TGT​CAT​CAA​TGA​TG GAG	2	6.0
	OFT2	5,852	chr8	TGA​AGT​TGT​CAT​CAA​TGA​TG GAG	2	6.0
	OFT3	91941448	chr9	TGA​AAT​TAT​GAT​CAA​TGA​TG GGG	3	2.7
	OFT4	31647797	chr23	ACA​TAT​TGT​CAT​CAA​TGA​TA CAG	3	1.9
	OFT5	65232401	chr9	TCA​ACT​TGT​AAT​CAA​TGA​TG CAG	4	1.5
	OFT6	30230581	chr1	AGA​ATT​TGA​AAT​CAA​TGA​TG GGG	3	1.5
	OFT7	51384676	chr3	ACA​AAT​TGT​AAG​CAA​TGA​TG TGG	3	1.4
	OFT8	ENSOARG00000017529	chr1	AGA​ACT​TGG​CAT​GAA​TGA​TG AAG	3	0.7
	OFT9	ENSOARG00000011021	chr21	TGA​AAG​TGT​CAT​CAG​TGA​TG GAG	3	0.6
	OFT10	ENSOARG00000018971	chr15	AAA​TAT​TGG​CCT​CAA​TGA​TG TGG	4	0.5
On target		IFNAR2	chr6	ACC​ACT​GAA​TTT​GTA​TCC​CA GGG	-	-
Off target	OFT11	144799426	chr1	AAC​ACT​GGT​TTT​GTA​TCC​CA GGG	3	1.7
	OFT12	464	chr5	GCA​CCT​GTA​TTT​GTA​TCC​CA GGG	4	1.5
	OFT13	82466814	chr5	AAT​GAT​GAA​TTT​GTA​TCC​CA AAG	4	1.3
	OFT14	39610637	chr12	CTC​CCT​GAA​TTT​GTA​TCC​CT GGG	4	0.9
	OFT15	108244924	chrX	ATC​CCT​GGA​TGT​GTA​TCC​CA GGG	4	0.9
	OFT16	57537802	chr4	ACA​TAT​GAT​TTT​GTA​TCC​CA GAG	4	0.9
	OFT17	54969806	chr5	GCC​TCT​GAA​GAT​GTA​TCC​CA AAG	4	0.9
	OFT18	20531845	chr6	ACT​ATT​GAA​AGT​GTA​TCC​CA TAG	4	0.8
	OFT19	99052667	chr5	AGC​TCT​GTA​TTT​ATA​TCC​CA GGG	4	0.7
	OFT20	44679365	chr10	TCC​TTT​GAA​TTT​ATA​TCC​CA AGG	4	0.7

### Genotyping of lambs produced by SCNT and breeding

**FIGURE 2:**
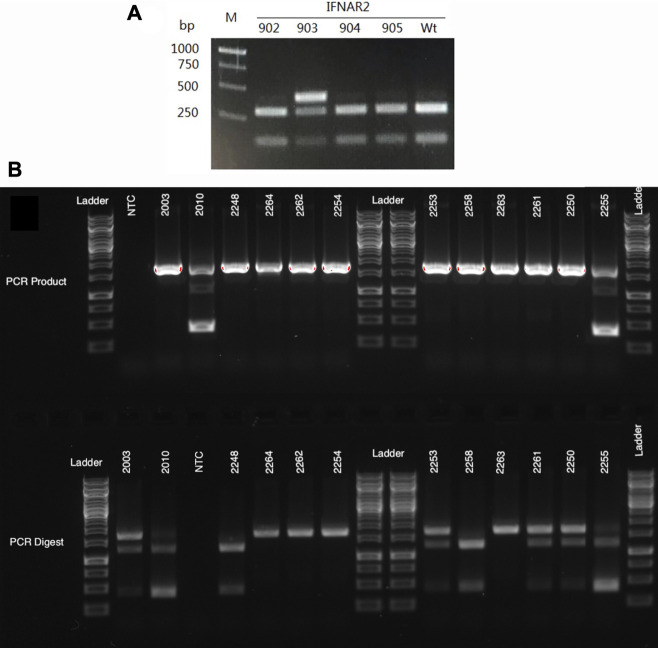
Genotyping gels for *IFNAR2* knockout sheep. **(A)** An example of the initial PCR-RFLP typing results for offspring of 7IFN2, an *IFNAR2* ^**−/−**^ ram. PCR amplification was done with primers created for screening the knockout cell colonies that amplify a 418 bp segment of the gene. Since the sire was a biallelic knockout and the dams were WT Romneys, the lambs were all expected to have one WT and one knockout allele. However, three of the four lambs (IFN902, 904 and 905) appeared to only have the WT allele, which is cut by the restriction enzyme NcoI. In contrast, lamb IFN903 had both a WT and an uncut allele with the previously identified 5 bp deletion. These results suggested that the sire carried a second *IFNAR2* knockout allele with a large deletion. **(B)** The top gel shows PCR amplification of genomic DNA with primers that amplify a 1,706 bp segment of the *IFNAR2* gene. Two of the sheep on this gel, IFN2010 and IFN2255, have a large 1,282 bp deletion, resulting in a much smaller 424 bp amplicon. The bottom gel shows the PCR products following digestion with NcoI. The WT allele, which is cut by NcoI, appears as two bands of 1,259 and 447 bp. The knockout allele with a small 6 bp deletion remains uncut and is seen as a 1,701 bp band.

The genotypes of all lambs produced by SCNT and breeding were confirmed using PCR-RFLP assays. The primers and restriction enzymes used for these analyses are listed in Tables 1, 2, respectively. In addition, PCR products from selected individuals were sequenced to confirm the sequence of the indels. When we typed the first cohort of lambs sired by ram 7IFN2, one of the *IFNAR2* ^***−/−***^ SCNT rams from colony #57, we discovered that 15 of the 28 lambs did not have a detectable knockout allele (Figure 2A). New primers further from the target site revealed that the lambs lacking the previously identified knockout allele with a 5 bp deletion had a 1,282 bp deletion. These primers were subsequently used to detect the WT and both knockout alleles in this line of sheep (Figure 2B), which is the line used to develop our *IFNAR2* ^**+/−**^ breeding herd described below.

### Phenotypic changes in IFNAR knockout sheep

Biallelic *IFNAR1* and *IFNAR2* knockout lambs were generally healthy at birth and nursed normally. While some biallelic knockout sheep have survived for long periods of time under farm conditions, the longest surviving individual being a ram who lived to about 4.5 years of age and produced over 80 offspring, 60% of our IFNAR knockout sheep born in 2017 through 2021 have died between 1 and 12 months of age due to viral, bacterial, or protozoal infections compounded by weight loss and emaciation. The most common necropsy findings were bronchopneumonia, ruminitis, abomacitis, enteritis and colitis, which are frequently initiated by a viral infection even when the cause of death is a bacterial or protozoal pathogen. There was a big difference in the death rate of male and female lambs during this critical period, for ram lambs the death rate was 0% (0/4 rams) while for ewe lambs it was 82% (9/11 ewes), which is a significant difference (*p* = 0.01 with Fisher’s Exact Test). Several ewe lambs died from unusual problems, including polioencephalomalacia and protozoal encephalitis (likely toxoplasmosis), and this may partially explain the high death rate. Consequently, we will need to see if the difference in male and female survival persists with the production of more *IFNAR2* ^***−/−***^ offspring.

**FIGURE 3:**
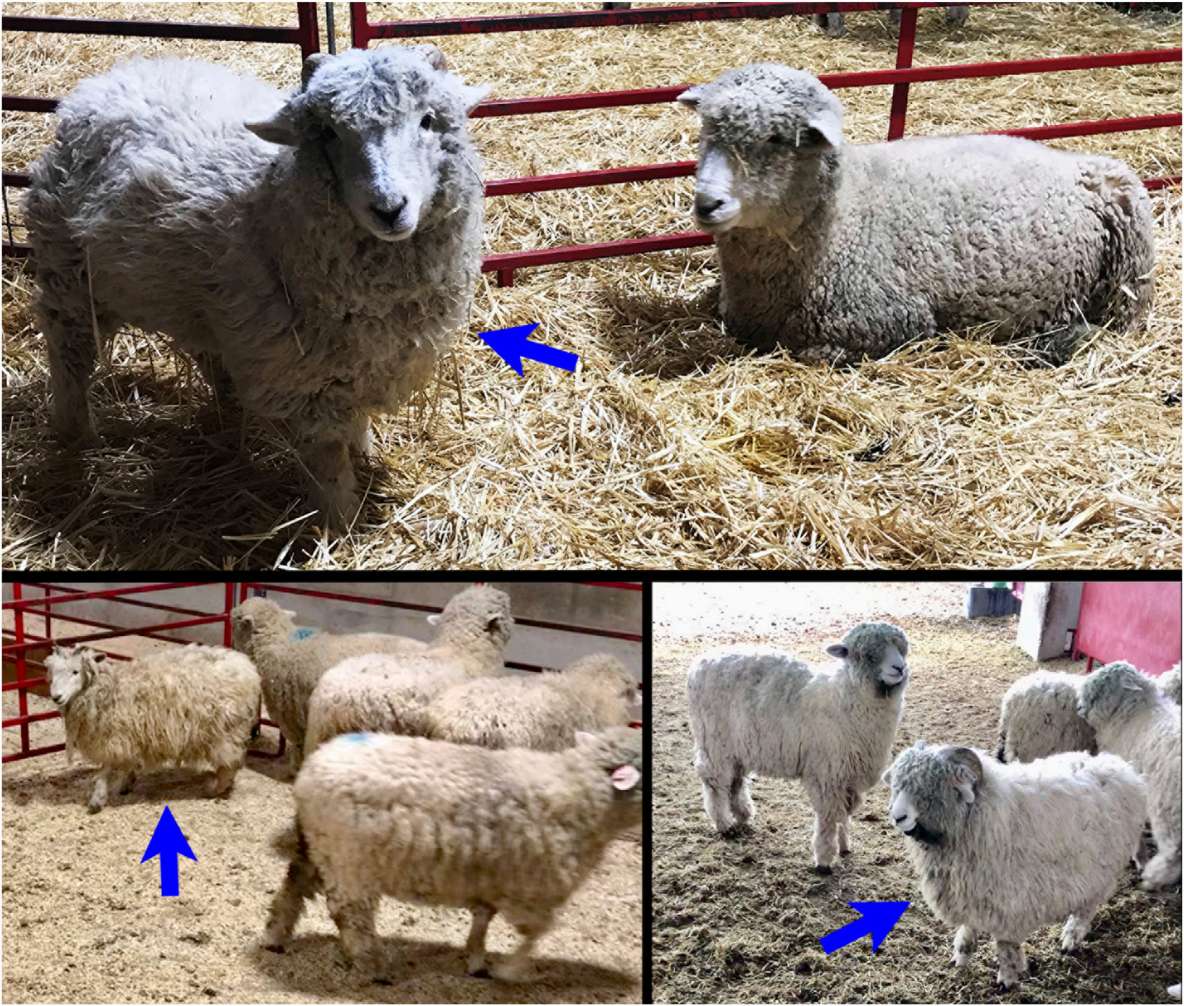
The top image shows a SCNT, *IFNAR1* ^**−/−**^ ewe born in 2016 at 12.5 months of age. The bottom images show two 8-month-old *IFNAR2* ^**−/−**^ sheep produced by breeding in 2021, a ewe (left) and a ram (right). All three *IFNAR* ^***−/−***^ sheep have abnormal wool, which looks more like the wool of a haired sheep, and horns, which are not normally present on Romney sheep. The *IFNAR* ^***−/−***^ sheep are indicated by blue arrows.

When the first IFNAR knockout sheep were born, we noticed that they had an abnormal haircoat. Their hair was long and straight, like the hair of haired sheep, and quite distinct from the wool of normal Romney sheep. All *IFNAR1* ^***−/−***^ and *IFNAR2* ^***−/−***^ sheep, whether produced by SCNT or by breeding, have exhibited this phenotype. In addition, the biallelic knockout sheep grow horns, while WT Romney sheep are polled (Figure 3). In contrast to the biallelic knockout sheep, their monoallelic *IFNAR1* or *IFNAR2* knockout relatives have a normal Romney phenotype.

### Assessment of the cellular response to IFNA

**FIGURE 4:**
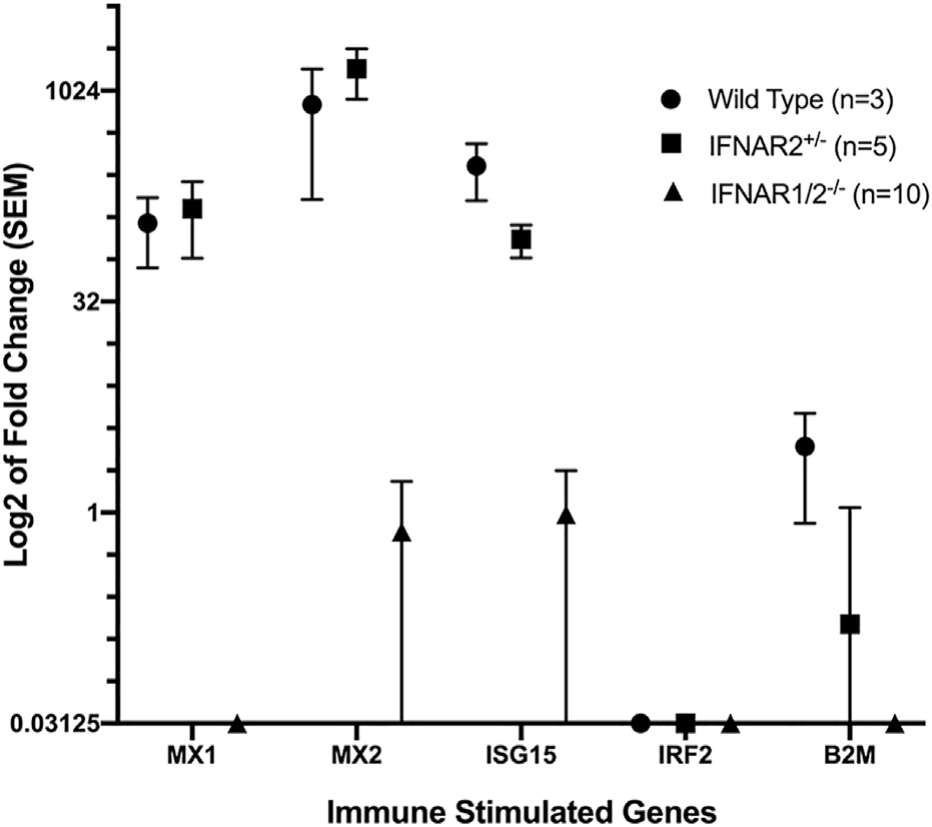
qRT-PCR analysis of fibroblast responses to human IFNA. Data were normalized using the average expression of four housekeeping genes. The *IFNAR1/2* ^***−/−***^ group (*IFNAR1* ^***−/−***^ and *IFNAR2* ^***−/−***^ sheep) differed significantly from WT and *IFNAR2* ^***+/−***^ sheep for *MX1*, *MX2* and *ISG15* (*p* < 0.01) using pairwise comparisons with Student’s *t*-test.

Type I IFNs activate the type I IFN receptor, IFNAR, and induce cells to upregulate numerous ISGs. To make sure that our IFNAR knockout sheep lacked functional type I IFN receptors, we developed a qRT-PCR assay for upregulation of ISGs by IFNA. WT and monoallelic knockout fibroblasts upregulated expression of three ISGs–MX1, MX2 and ISG15–but did not upregulate expression of IRF2 or B2M (Figure 4). Fibroblasts from biallelic knockout sheep did not upregulate expression of ISGs in response to IFNA, confirming their lack of functional type I IFN receptors.

### Examination of the functional role of IFNAR in antiviral defense, both *in vitro* and *in vivo*

**FIGURE 5:**
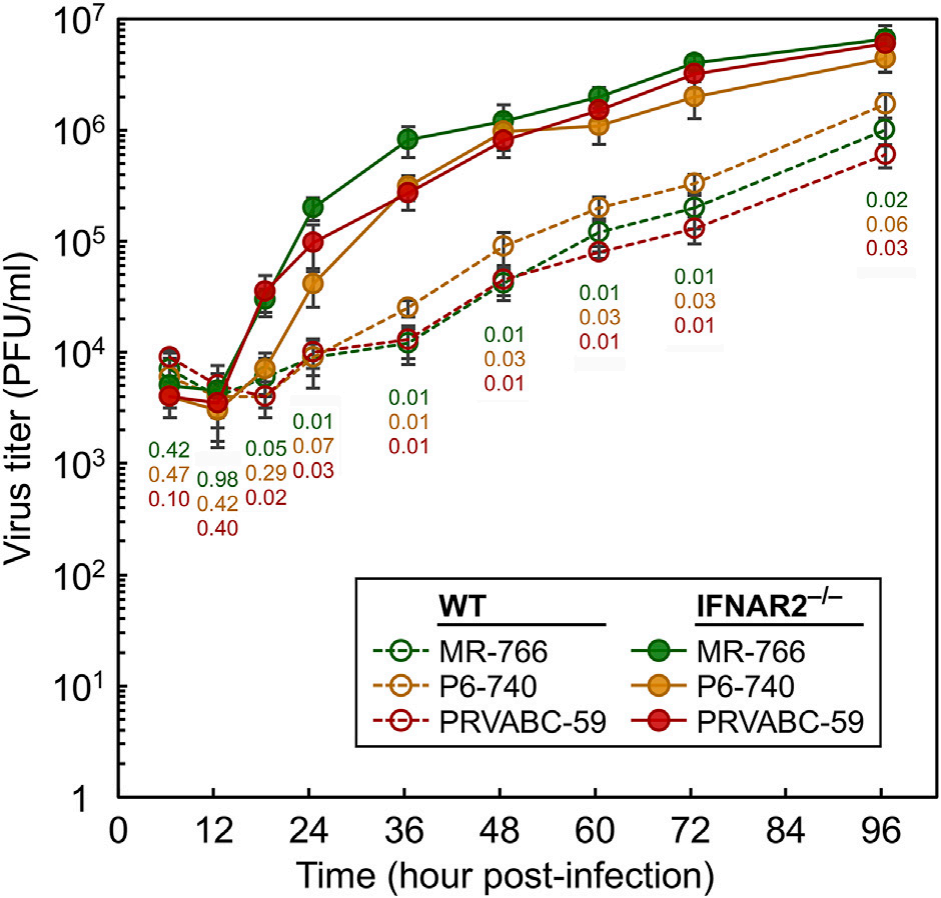
Comparison of the replication kinetics of ZIKV in SFFs isolated from WT and *IFNAR2* ^***−/−***^ sheep. Primary fibroblasts derived from WT and *IFNAR2* ^***−/−***^ sheep fetuses were infected at a multiplicity of infection of one with each of the following three strains of ZIKV: MR-766, P6-740, and PRVABC-59. At the indicated time points after infection, cell culture supernatants were harvested to determine the yield of progeny virions using a standard plaque assay on Vero cells. The virus titers in plaque-forming units per ml (PFU/ml) are plotted as a line graph of the average with standard deviation of two replicates. Statistical comparisons were done using a two-tailed *t*-test at each time point for each viral strain produced from WT and *IFNAR2* ^*−/−*^ fibroblasts. *p*-values are color-coded: green (MR-766), orange (P6-740), and red (PRVABC-59).

We first examined the potential antiviral role of IFNAR2 *in vitro*, by comparing the ability of primary fibroblasts isolated from WT and *IFNAR2* ^**−/−**^ fetuses to inhibit the replication of ZIKV, a zoonotic flavivirus that can infect both humans and animals and cause a variety of neurological diseases (Song et al., 2017; Yun and Lee, 2017). To this end, primary fibroblasts derived from WT and *IFNAR2* ^**−/−**^ fetuses were infected at a multiplicity of infection of one with each of three genetically distinct ZIKV strains that represent the full range of geographical and temporal diversity among isolates, namely African MR-766 (Uganda, 1947), Asian P6-740 (Malaysia, 1966), and American PRVABC-59 (Puerto Rico, 2015; Yun et al., 2016a; Yun et al., 2018). Following infection, the amount of progeny virions released into the culture medium was quantitated over a period of 4 days by a standard plaque assay. With all three ZIKV strains, we found that, regardless of the viral strain, the virus was able to grow in both *IFNAR2* ^**−/−**^ and WT cells but replicated more efficiently in *IFNAR2* ^**−/−**^ than WT cells, producing about 1-2 log higher virus titers in the supernatants starting from 18 h after infection until the end of the experiment when the maximum virus titers of 4.4–6.6 × 10^6^ PFU/ml were reached (Figure 5). Thus, these results demonstrate that IFNAR plays an important role in the ovine antiviral response against ZIKV infection in cell culture.

**FIGURE 6:**
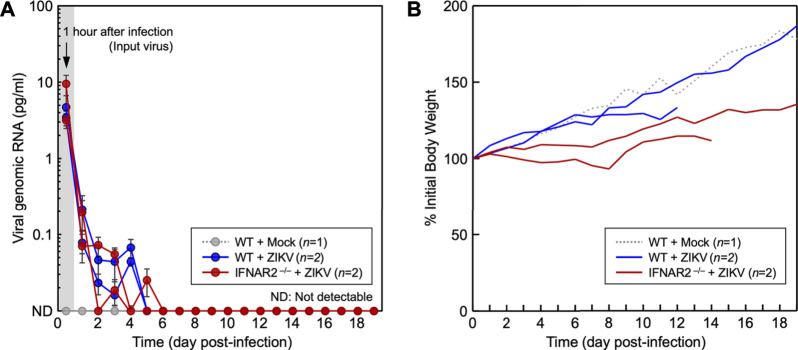
Study of ZIKV replication in WT and *IFNAR2* ^**−/−**^ lambs. One-month-old WT (*n* = 2) and *IFNAR2* ^**−/−**^ (*n* = 2) ewe lambs were each inoculated intravenously with 2.0 × 10^6^ PFU of ZIKV PRVABC-59. As a control, an age- and sex-matched WT lamb was mock-infected. **(A)** The amount of viral genomic RNA, an indication of viral replication, was quantitated by qRT-PCR using the blood specimens taken daily for 19 days after infection. The amount of viral genomic RNA detected at 1-h post-infection serves as a reference for the viral inoculum. The amount of viral genomic RNA for each sample was determined based on a standard curve generated using a full-length infectious cDNA clone of ZIKV PRVABC-59. **(B)** Body weights were recorded daily and plotted as the changes in body weight relative to the initial value at day zero.

Next, we evaluated the functional role of IFNAR2 in inhibiting ZIKV replication *in vivo* by conducting animal infection experiments. In the first experiment, two 1-month-old *IFNAR2* ^**−/−**^ ewe lambs and two age-matched WT control ewe lambs were each infected intravenously with 2.0 × 10^6^ PFU of ZIKV PRVABC-59. As an indication of ZIKV replication, the amount of virus present in blood samples withdrawn daily for 19 days after infection was quantitated by qRT-PCR to detect viral genomic RNA using a fluorogenic probe specific for the ZIKV NS3 protein-coding region. Our data showed that the initial viral inoculum detected at 1 h post-infection was decreased rapidly to undetectable levels within the first 5–6 days after infection of both *IFNAR2* ^**−/−**^ and WT lambs, with no signs of viral amplification (Figure 6A). Also, no clinical signs of ZIKV infection were observed in both groups. However, the body weight gain of two infected *IFNAR2* ^**−/−**^ lambs was significantly lower than that of two infected WT lambs (Figure 6B). One of the WT lambs was euthanized on day 13 because of a catheter infection involving an unknown microorganism and one IFNAR2^**−/−**^ lamb was euthanized on day 15 because she was unable to stand due to profound weakness. At necropsy, both *IFNAR2* ^**−/−**^ lambs had viral abomasitis, enteritis, and lymphadenitis; however, they tested negative for bovine viral diarrhea virus, blue tongue virus, coronavirus, and rotavirus, all of which are commonly associated with those complications in sheep. In addition, ZIKV was not detected by immunohistochemistry with a rabbit antiserum specific to the ZIKV NS1 protein or by RT-PCR with a pair of primers specific for the ZIKV NS3 protein-coding region. In the second experiment, we used a pair of 11-month-old *IFNAR2* ^**−/−**^ and WT rams, each infected with 2.0 × 10^7^ PFU of ZIKV PRVABC-59. This second experiment also showed similar results as seen in the first experiment (data not shown). Our data indicate that ZIKV replication is controlled efficiently not only in WT sheep but also in *IFNAR2* ^**−/−**^ sheep. Moreover, our results suggest that the lack of type I IFN signaling is presumably compensated by other types of IFN or other antiviral innate immune mechanisms.

## Development of an *IFNAR2* ^*+/−*^ breeding herd

In 2018, 25 WT Romney ewes were bred to 7IFN2, an 18-month-old *IFNAR2* ^**−/−**^ ram. Twenty-two ewes were confirmed pregnant, an 88% pregnancy rate, and 28 *IFNAR2* ^**+/−**^ lambs (16 rams and 12 ewes) were produced (1.3 lambs/ewe). Table 6 is a summary of the *IFNAR* knockout sheep produced by both cloning and breeding from 2017 through 2022. We presently have a breeding herd of 32 *IFNAR2* ^**+/−**^ ewes that are being bred to *IFNAR2* ^**−/−**^ or *IFNAR2* ^**+/−**^ rams to produce *IFNAR2* ^**−/−**^ and *IFNAR2* ^**+/−**^ lambs. The *IFNAR2* ^**+/−**^ lambs and *IFNAR2* ^**−/−**^ ram lambs are used as replacements for the breeding herd, while the *IFNAR2* ^**−/−**^ ewe lambs are being used for breeding experiments.

**TABLE 6:** Production of *IFNAR* knockout sheep.

Year	Number of biallelic knockout lambs from cloning	Number of monoallelic knockout lambs from breeding	Number of biallelic knockout lambs from breeding
2017	4 (3♂^a^/1♀^b^)	**—**	**—**
2018	5 (0♂/5♀^a^)	1 (0♂/1♀^a^)	**—**
2019	3 (0♂/3♀^a^)	28 (16♂^a^/12♀^a^)	**—**
2020	**—**	27 (13♂^a^/14♀^a^)	**—**
2021	**—**	6 (3♂^a^/3♀^a^)	10 (2♂^a^/8♀^a^)
2022	**–**	14 (7♂^a^/7♀^a^)	10 (7♂^a^/3♀^a^)

^a^ *IFNAR2* knockouts.

b *IFNAR1* knockouts.

## Breeding trial with an *IFNAR1* ^*−/−*^ Ewe

At 17 months of age the sole *IFNAR1* ^**−/−**^ ewe born in 2017 underwent estrus synchronization and was placed with a WT ram from September 10–24, 2018. She was marked (bred) by the ram on September 12th. Unfortunately, this ewe died of pneumonia on November 17 when her fetus would have been about 65 days old. There was no evidence of a fetus at necropsy, suggesting that this *IFNAR1* ^**−/−**^ ewe was infertile.

## Discussion

We successfully established biallelic *IFNAR1* and *IFNAR2* knockout SFF cell lines and produced lambs from seven different knockout cell lines by SCNT (Table 4). Biallelic *IFNAR1* or *IFNAR2* knockout lambs were easily recognized by their unique hair phenotype (Figure 3). In addition, all biallelic knockout lambs tested were functional IFNAR knockouts since fibroblast cells from these lambs failed to respond to IFNA by upregulating expression of ISGs (Figure 4). All but one group of lambs had the expected genotype when genotyped with the PCR-RFLP assay(s) used to screen the corresponding SFF cell line. The one exception was two lambs from the same cell line that were predicted to be biallelic knockouts for both *IFNAR1* and *IFNAR2* but turned out to only be monoallelic knockouts for *IFNAR1* (Table 4). Nevertheless, these lambs were functional knockouts because both copies of *IFNAR2* were inactivated.

When we bred one of our initial *IFNAR2* ^**−/−**^ rams to a group of WT Romney ewes to produce monoallelic knockout offspring, we were surprised to find that half of the offspring lacked the expected knockout allele with a 5 bp deletion. After further investigation, we found that these lambs had inherited a knockout allele with a large, 1,282 bp deletion that was not detected with the initial screening assay (Figure 2). We have found large deletions in other sheep knockout cell lines. This phenomenon has also been observed in mice after CRISPR/Cas9-mediated editing (Parikh et al., 2015; Shin et al., 2017; Adikusuma et al., 2018). Together, our data and the mouse data suggest that large deletions may happen fairly frequently.

The long and straight haircoat of *IFNAR1* ^**−/−**^ and *IFNAR2* ^**−/−**^ Romney sheep, a breed that normally has short and woolly hair, is intriguing. It is not clear why loss of type I IFN signaling results in a change in hair phenotype. However, modern domestic sheep were selected for short and woolly fleece while ancestral breeds had long and hairy fleece. The change from long and hairy to short and woolly fleece was recently mapped to the *IFN regulatory factor 2 binding protein 2* (*IRF2BP2*) gene, which is a transcriptional repressor of IFN regulatory factor 2 (IRF2) expression (Demars et al., 2017; Devon Fitzpatrick, personal communication). Demars et al. (2017) found that the naturally occurring “woolly” *IRF2BP2* allele of domestic sheep has an antisense *EIF2S2* retrogene inserted in the 3’ UTR of *IRF2BP2*. This change results in formation of a double-stranded RNA construct that alters IRF2BP2 expression. Decreased expression of IRF2BP2 would result in increased expression of the transcription factor IRF2 and altered expression of type I interferons such as IFNA and IFNB (Taniguchi et al., 2001; Ramalho-Oliveira et al., 2019; Pastor et al., 2021). The findings of Demars et al. (2017) are consistent with our observations; together they suggest that a high level of type I IFN signaling results in a woolly phenotype and a low level of type I IFN signaling results in a hairy phenotype.

The potential antiviral role of IFNAR2 was investigated *in vitro*, by comparing the ability of primary fibroblasts isolated from WT and *IFNAR2* ^**−/−**^ fetuses to inhibit the replication of ZIKV (Figure 5). Although the three ZIKV strains tested were able to grow in both *IFNAR2* ^**−/−**^ and WT cells, they replicated more efficiently in *IFNAR2* ^**−/−**^ cells, producing about 1-2 log higher virus titers between 18 h and 96 h post-infection. These results demonstrate that ovine IFNAR plays an important role in the *in vitro* antiviral response against ZIKV.

An *in vivo* ZIKV infection experiment in 1-month-old IFNAR knockout ewe lambs showed that the initial viral inoculum detected at 1 h post-infection decreased rapidly to undetectable levels within the first 5–6 days after infection with no signs of viral amplification in either WT or *IFNAR2* ^**−/−**^ lambs, (Figure 6A). In addition, neither group exhibited clinical signs of ZIKV infection. However, the two infected *IFNAR2* ^**−/−**^ lambs gained much less weight during the trial than the WT lambs (Figure 6B). This suggests that these lambs were expending more energy to control their ZIKV or other infections. Although no lesions attributable to ZIKV were identified at necropsy, both *IFNAR2* ^**−/−**^ lambs had gastrointestinal tract lesions (ruminitis, abomasitis, enteritis, colitis, and lymphadenitis) suggestive of an enteric viral infection. Surprisingly, ZIKV was not detected in any of the tissues from the lambs by either immunohistochemistry or RT-PCR. These results suggest that both WT and *IFNAR2* ^**−/−**^ sheep can efficiently control ZIKV replication, which differs from other published studies (Schwarz et al., 2019; Schwarz et al., 2020).

The results from our ZIKV infection studies and the observation that some IFNAR knockout sheep survive for years under farm conditions, suggest that sheep can compensate for a lack of type I IFN signaling by relying on other types of IFN or other innate antiviral mechanisms. Sheep have two additional types of IFN, the type II and type III interferons. IFN-gamma (IFNG), the sole type II IFN, is an important regulatory and effector cytokine of the adaptive immune response (Selinger and Reinis, 2018). Type III IFNs are a group of related molecules of the IFN-lambda (IFNL) family. The IFNL family of cytokines are specialized for protecting mucosal tissues against viral infections (Kotenko et al., 2019; Vlachiotis and Andreakos, 2019; Ye et al., 2019; Broggi et al., 2020). Type III IFNs were discovered in 2003 and have functions that partially overlap those of type I IFNs (Diaz-San Segundo et al., 2011; Lazear et al., 2019; Stanifer et al., 2019). Each type of IFN has a unique receptor: IFNAR, IFNGR and IFNLR, respectively (Mesev et al., 2019). We hypothesize that the IFNAR knockout sheep were able to survive a ZIKV challenge and can survive under farm conditions because of the ability of IFNG and IFNL to partially compensate for the absence of IFNAR signaling.

While some IFNAR knockout sheep have remained healthy under farm conditions for several years, the high death rate during the first year of life is of concern both from the standpoint of animal welfare, and because it makes it difficult to produce mature animals for breeding studies. While it was important to find out how these sheep do under normal husbandry conditions, it is imperative that we improve the welfare of these sheep. Most lambs that died during the first year of life, died from bronchopneumonia between two and 6 months of age, which is when passive immunity is waning and the adaptive immune system is still relatively naïve. Consequently, we are now monitoring the lambs very closely during this time and treating them with long-acting antibiotics at the first sign of respiratory distress. We are also separating the biallelic knockout lambs from the other lambs to decrease competition for feed and exposure to pathogens.

Ruminants utilize a type I IFN, IFN-tau (IFNT), and the type I interferon receptor (IFNAR), for pregnancy recognition (Roberts et al., 2016; Yoshinaga, 2018). Therefore, IFNAR knockout sheep are a valuable model for studying pregnancy recognition in ruminants. In addition, it has been suggested that IFNT also has autocrine effects on trophoblast cells that promote conceptus elongation (Brooks and Spencer, 2015; Hansen et al., 2017). An autocrine role for IFNT is consistent with the expression of IFNAR on trophoblast cells (Han et al., 1997; Imakawa et al., 2002). However, *in vivo* morpholino antisense oligonucleotide loss-of-function experiments did not support a direct autocrine effect (Brooks and Spencer, 2015; Hansen et al., 2017). We have shown that *IFNAR1* ^***−/−***^ and *IFNAR2* ^***−/−***^ conceptuses produced by SCNT or breeding develop normally in WT ewes. Production of a substantial number of *IFNAR1* ^***−/−***^ and *IFNAR2* ^***−/−***^ sheep proves that elongation of the conceptus does not depend on a direct autocrine response involving IFNT and IFNAR (Brooks and Spencer, 2015; Hansen et al., 2017). Either IFNL induces a redundant response, there is an alternative receptor for IFNT, or IFN signaling is not required. Furthermore, the SCNT pregnancy rates for IFNAR knockout embryos during the initial two breeding seasons (67% in 2016 and 69% in 2017) were unprecedented; a typical pregnancy rate for SCNT in sheep is 30%–40%. These high pregnancy rates suggest that IFNAR knockout embryos may have increased embryonic survival. In previous studies with ruminants, SCNT was associated with increased expression of major histocompatibility complex class I (MHC-I) proteins on trophoblast cells, accumulation of T lymphocytes in the endometrium, and immune-mediated rejection of the conceptus (Hill et al., 2002; Rutigliano et al., 2016; Rutigliano et al., 2017; Koroghli et al., 2018). Furthermore, MHC-I expression in bovine trophoblast cells was correlated with the expression of transcription factors (IRF1, CIITA and STAT1), which are induced by type I IFN, IFNG and IFNL (Shi et al., 2018). In addition to upregulating MHC-I expression, these transcription factors induce expression of several inflammatory mediators (Hansen et al., 2017; Niedzwiedzka-Rystwej et al., 2017). Elimination of trophoblast IFNAR expression would abolish any response to type I IFNs. Consequently, we hypothesize that loss of IFNAR might decrease the inflammatory crosstalk between the conceptus and maternal immune system and promotes embryonic and fetal survival.

Our successful production of a breeding herd of *IFNAR2* ^**+/−**^ ewes by breeding an *IFNAR2* ^**−/−**^ ram produced by SCNT to WT Romney ewes has established that *IFNAR2* ^**−/−**^ rams are fertile. We have also established that *IFNAR2* ^**+/−**^ ewes are fertile as over 20 *IFNAR2* ^**+/−**^ ewes have had successful pregnancies (Table 6). To date, only one *IFNAR1* ^**−/−**^ ewe has reached sexual maturity. This ewe was bred but failed to become pregnant. This is consistent with the established paradigm that IFNT signaling via IFNAR is required for the establishment of pregnancy in ruminants. Nevertheless, more data is needed to prove the hypothesis that *IFNAR2* ^***−/−***^ ewes will be sterile due to an inability to respond to IFNT. If *IFNAR2* ^***−/−***^ ewes are infertile, it is also important to determine if IFNT signaling is required for anything else in addition to rescuing the CL. If the only essential function for IFNT is to rescue the CL, then it should be possible to establish pregnancies in IFNAR knockout ewes by supplementing them with progesterone.

## Conclusion

We have established a unique IFNAR knockout sheep model that can be used for studying both the pathogenesis of viral infections and pregnancy recognition in sheep. These sheep are more susceptible to infectious diseases but can survive under farm conditions. Establishment of a breeding herd of *IFNAR2* ^**+/−**^ ewes has greatly facilitated production of *IFNAR2* ^***−/−***^ sheep for infectious disease and reproductive studies.

## Data Availability

The original contributions presented in the study are included in the article/Supplementary Material, further inquiries can be directed to the corresponding authors.
