# Supplementary material for: Development and characterization of type I interferon receptor knockout sheep: A model for viral immunology and reproductive signaling
Source: Front Genet. 2022 Sep 14;13:986316. doi: 10.3389/fgene.2022.986316 (PMC9556006; doi:10.3389/fgene.2022.986316)
Supplement: Supplementary file 1 [file Table1.pdf]

Supplemental Table 1: Primers for off-target analysis.

Off target	Forward (5'->3')	Reverse (5'->3')	TM (°C)	Product size (bp)
OFT1	GACTGAGAGAGGAGAGTCAGAC	GTCTGCAGGAAACTTCTCAACA	60.8	627
OFT2	GTCTGCAGGAAACTTCTCAACA	ATGTGACTAGCTGGATCCAAGT	60.8	685
OFT3	AAGGCATTCTTCTCTGAGCATG	GATTTCTCAAGAGGCAGGTCAG	62.7	499
OFT4	GGCTGTTGTATGGCATCACATA	TGAGCCACCAGGATTAATGAGA	60.8	572
OFT5	ACCAATTGACTCTCAGTCGAGA	ACTCTGAAGTCACAGGTCAGAA	60.8	428
OFT6	ACCTCACATTACCTTCTCCTCC	AACTACAACAAAGGGAGGCAAG	62.7	476
OFT7	AACCGGGAAACATTTAAGGGTC	GTCCCTTATCTTCAAGAGCTGC	60.8	404
OFT8	AGCTGTTCTCTGTTTGTTGTGT	AAAAACACAGCCAAAGCAATCC	58.9	696
OFT9	GGAGAGACCAGAGGAATCAGAA	CTTGAAGCAAGGCCAGTGATAT	60.8	705
OFT10	ACTATCCTCACAAATCCCGTCA	TTCACCCCATAGACAACAGGAT	60.8	485
OFT11	AAGTGTTGACAGATCTTGGCAA	ACTTGTGCTGCTCAAAATGTTG	58.9	361
OFT12	CTCTACCGTACCAAACACACTG	CTTTGAAGATGGAGGAGGATGC	62.7	425
OFT13	TTCAGACAAAGAAGCCCAAGAC	TACCATCTGAGCCATGAAGGAA	60.8	739
OFT14	AGACCCAGCACAACCAAATAAG	TGTGGGCTTTGGACTACATTTC	60.8	294
OFT15	TACTATGGATGACTGACCACCC	GAACATGCTCTTTAGTCCAGGG	62.7	437
OFT16	TGTCTCATCCTGGTGTGTTTTC	ACCAAATGGTCAGACCTCAAAC	60.8	259
OFT17	TTATCCAACCAAGGCAAACTGT	AGTGCCATGTTTAATGTGCTCA	58.9	384
OFT18	GGAAGAGACAAAGGGAAAGCAA	AGAGTCAGACACAACTGAGTGA	60.8	320
OFT19	GCACAAAAGGATTTCATTCGGG	CATCCGTCCATGTAATAGCCTG	60.8	576
OFT20	TATCCCACTCTTGCTGGTGTTA	GAAGTGGGTCCTTGTGCATTTA	60.8	474
