# Supplementary material for: Development and characterization of type I interferon receptor knockout sheep: A model for viral immunology and reproductive signaling
Source: Front Genet. 2022 Sep 14;13:986316. doi: 10.3389/fgene.2022.986316 (PMC9556006; doi:10.3389/fgene.2022.986316)
Supplement: Supplementary file 2 [file Table2.pdf]

Supplemental Table 2: Housekeeping and ISG primers used for qRT-PCR.

**Housekeeping Genes**		
**Gene**	**Forward Primer** (5'->3')	**Reverse Primer** (5'->3')
GAPDH	GGAGAAGGCTGGGGCTCAC	TGCTGACAATCTTGAGGGTATTGT
ACTB	GGCCAACCGTGAGAAGATGA	CCAGAGGCGTACAGGGACAG
YWHAZ	AGGTGCTGAGAAAAAACAGCAGA	CGTTTGGGATCAAGAACTTTTCC
EIF4A1	GGATTGGCTCACCGAGAAGAT	CCAGTAGGTCAGTGGTAATCAATACTCTG
**Interferon Stimulated Genes (ISGs)**		
**Gene**	**Forward Primer** (5'->3')	**Reverse Primer** (5'->3')
MX1	TCCCGACTGTTTACCAAAGTACGA	GTCCACAGCAGGCTCTTCCA
MX2	AGTTTCCTCCATCGTTGAGATTG	CATCATGGCTTTCTGCACCTTAT
IRF2	GTATGCGGTCCTGACTTCAACTATAA	CGGTGGTCACCTCCACAACT
ISG15	CATGATGGTATCCGAGCTGAAG	CACCTCCCTGCTGTCAAGGT
B2M	GCCAGAAGATGGAAAGCCAA	TACTGATCCTTGCTGTTGGGAG
