# Supplementary material for: Development and characterization of type I interferon receptor knockout sheep: A model for viral immunology and reproductive signaling
Source: Front Genet. 2022 Sep 14;13:986316. doi: 10.3389/fgene.2022.986316 (PMC9556006; doi:10.3389/fgene.2022.986316)
Supplement: Supplementary file 3 [file Table3.pdf]

Supplemental Table 3: Characterization of SFF colonies following targeted disruption of both *IFNAR1* and *IFNAR2*.

Cell line (Sex)	Targeted Genes	No. of Colonies Isolated	Biallelic Disruption of *IFNAR1* (%)	Biallelic Disruption of *IFNAR2* (%)	Biallelic Disruption of Both Genes (%)
SFF3 (F)	*IFNAR1* and *IFNAR2*	268	21 (7.8)	--	8 (3.0)
SFF5 (M)	*IFNAR1* and *IFNAR2*	139	--	31 (22.3)	18 (12.9)
