# Supplementary material for: Development and characterization of type I interferon receptor knockout sheep: A model for viral immunology and reproductive signaling
Source: Front Genet. 2022 Sep 14;13:986316. doi: 10.3389/fgene.2022.986316 (PMC9556006; doi:10.3389/fgene.2022.986316)
Supplement: Supplementary file 4 [file DataSheet1.PDF]

A) Sheep 7IFN3, *IFNAR1*$^{-/-}$

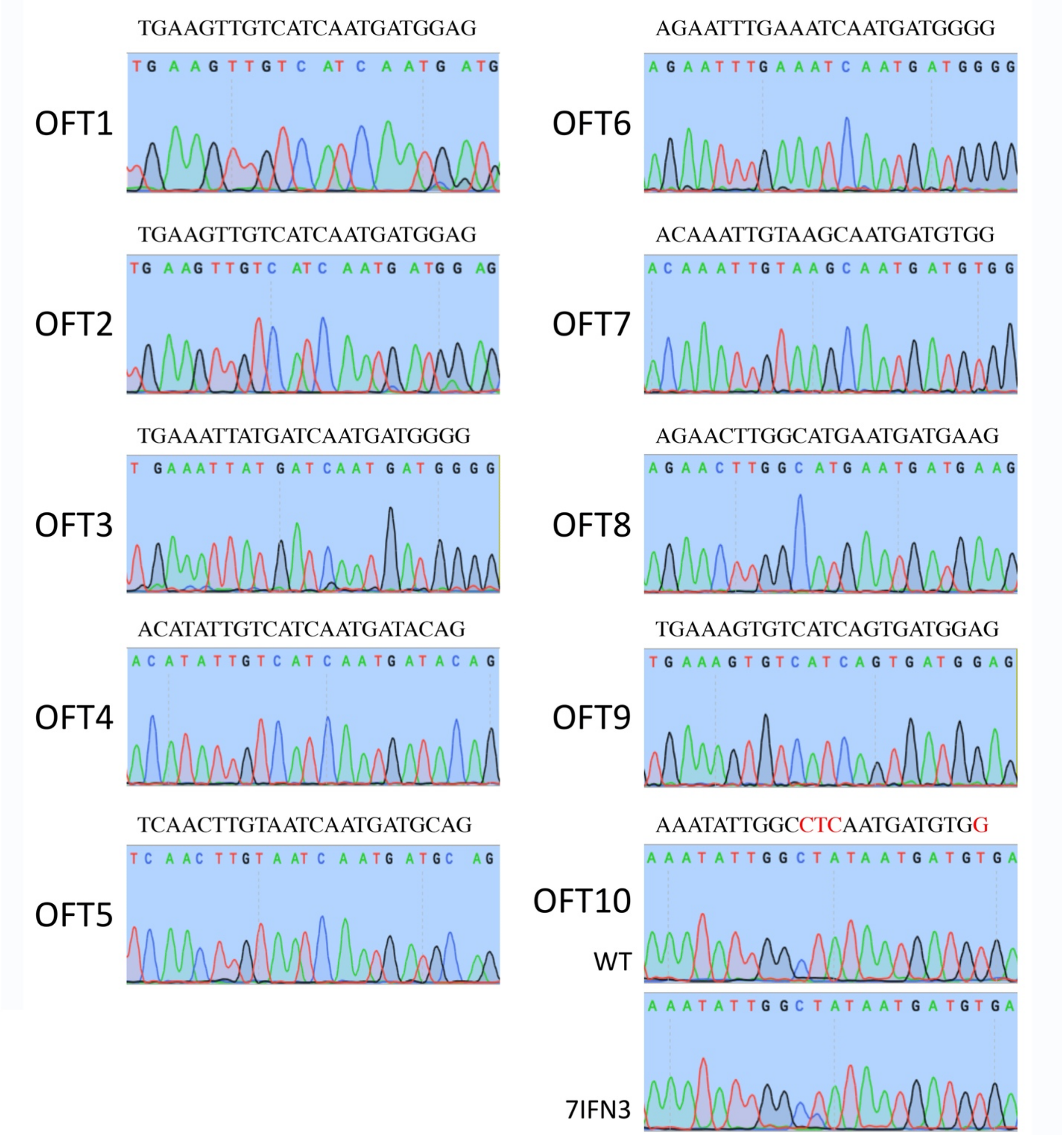

B) Sheep 7IFN1, *IFNAR2*$^{-/-}$

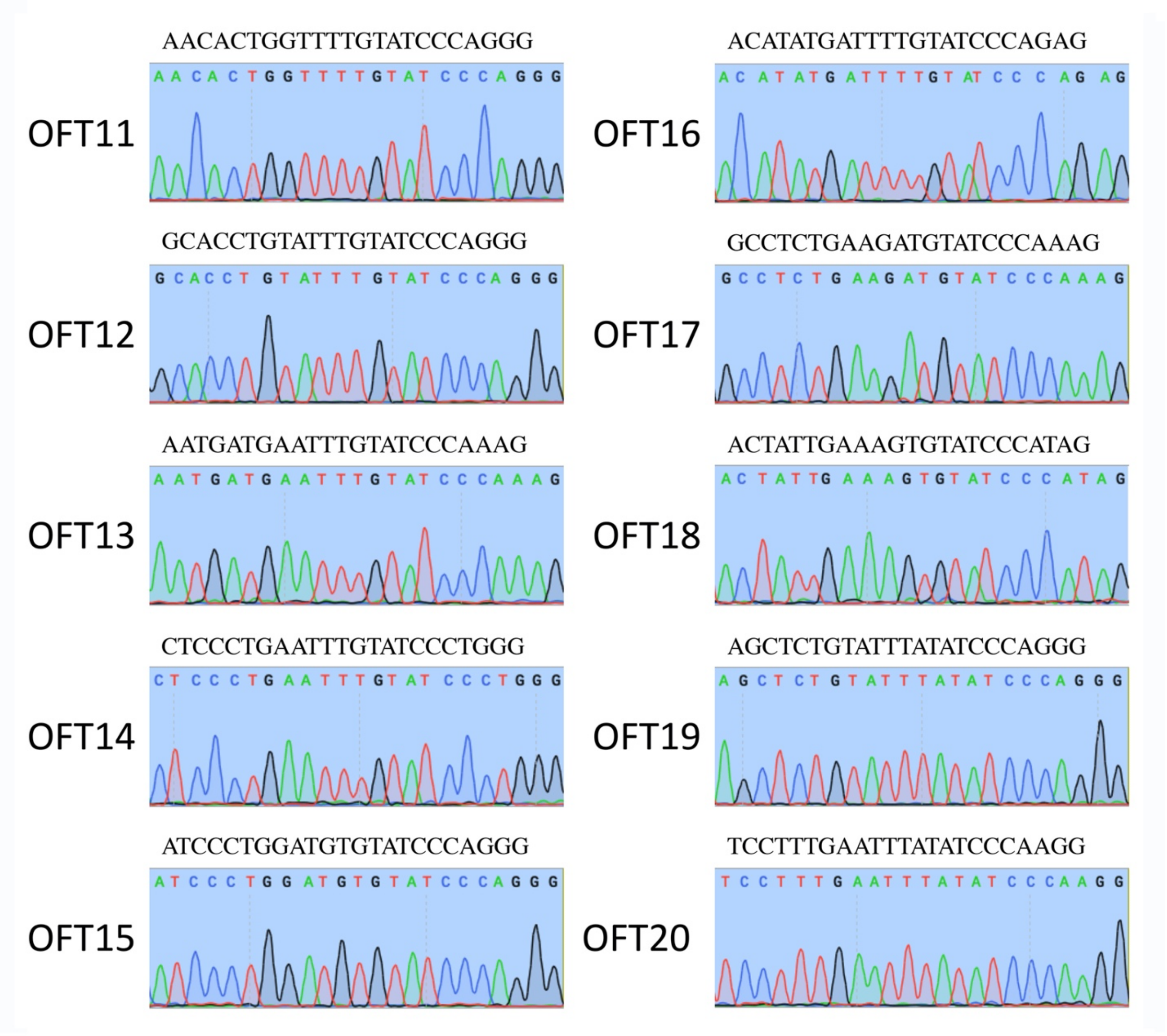

C) Sheep 7IFN2, *IFNAR2*$^{-/-}$

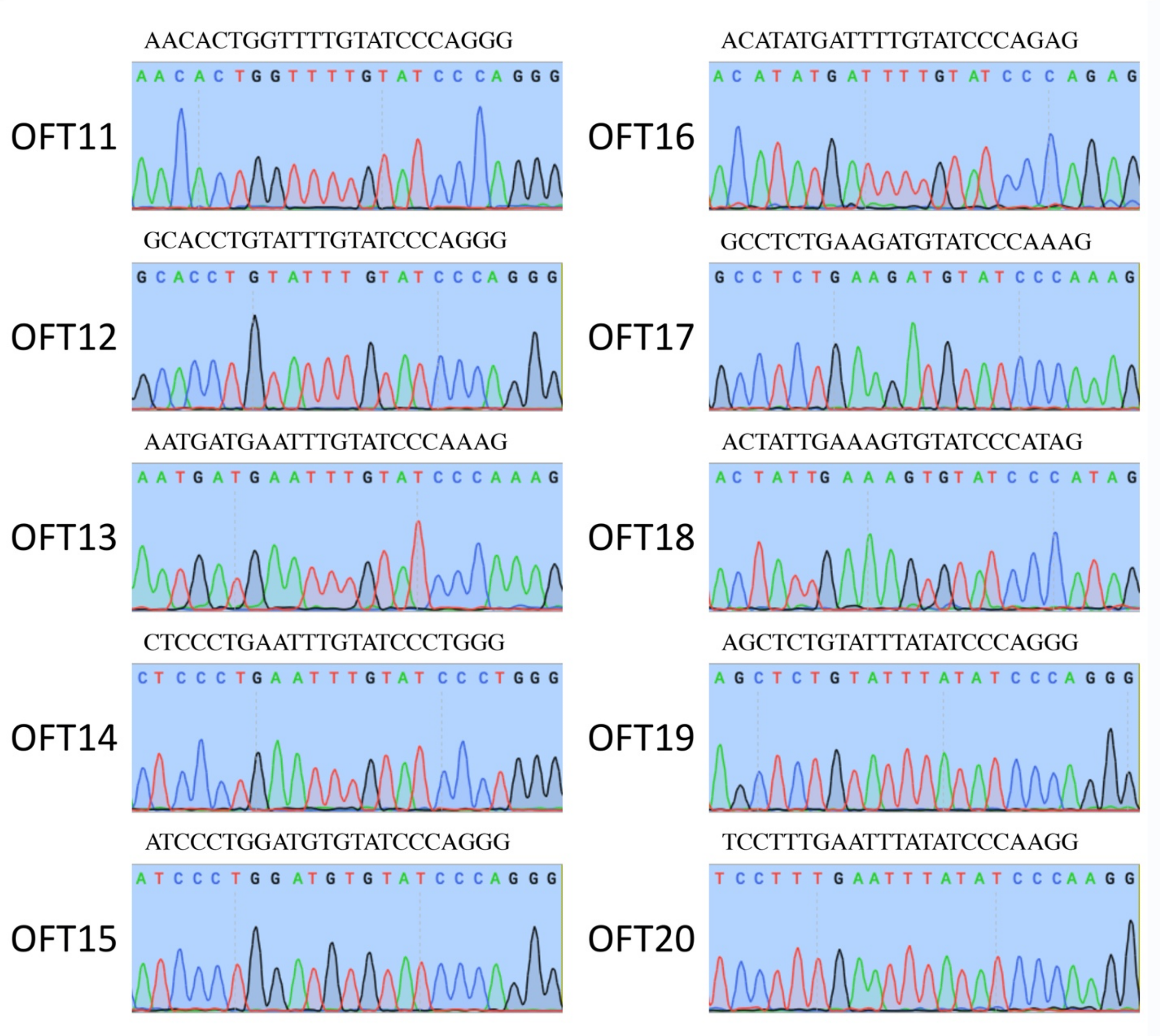

D) Sheep 7IFN4, *IFNAR2*$^{-/-}$

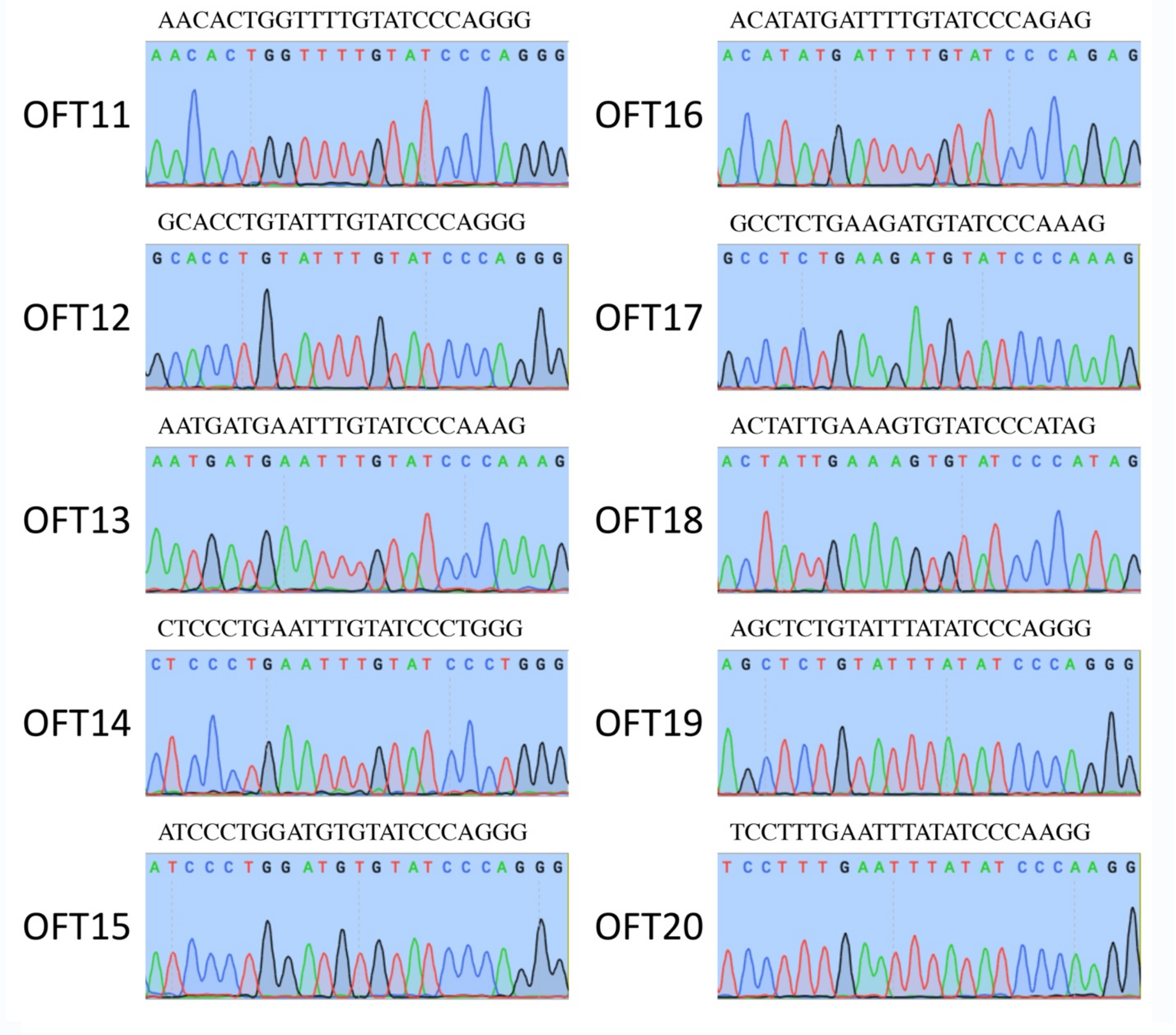

E) Sheep 7IFN-fetus, *IFNAR2*$^{-/-}$

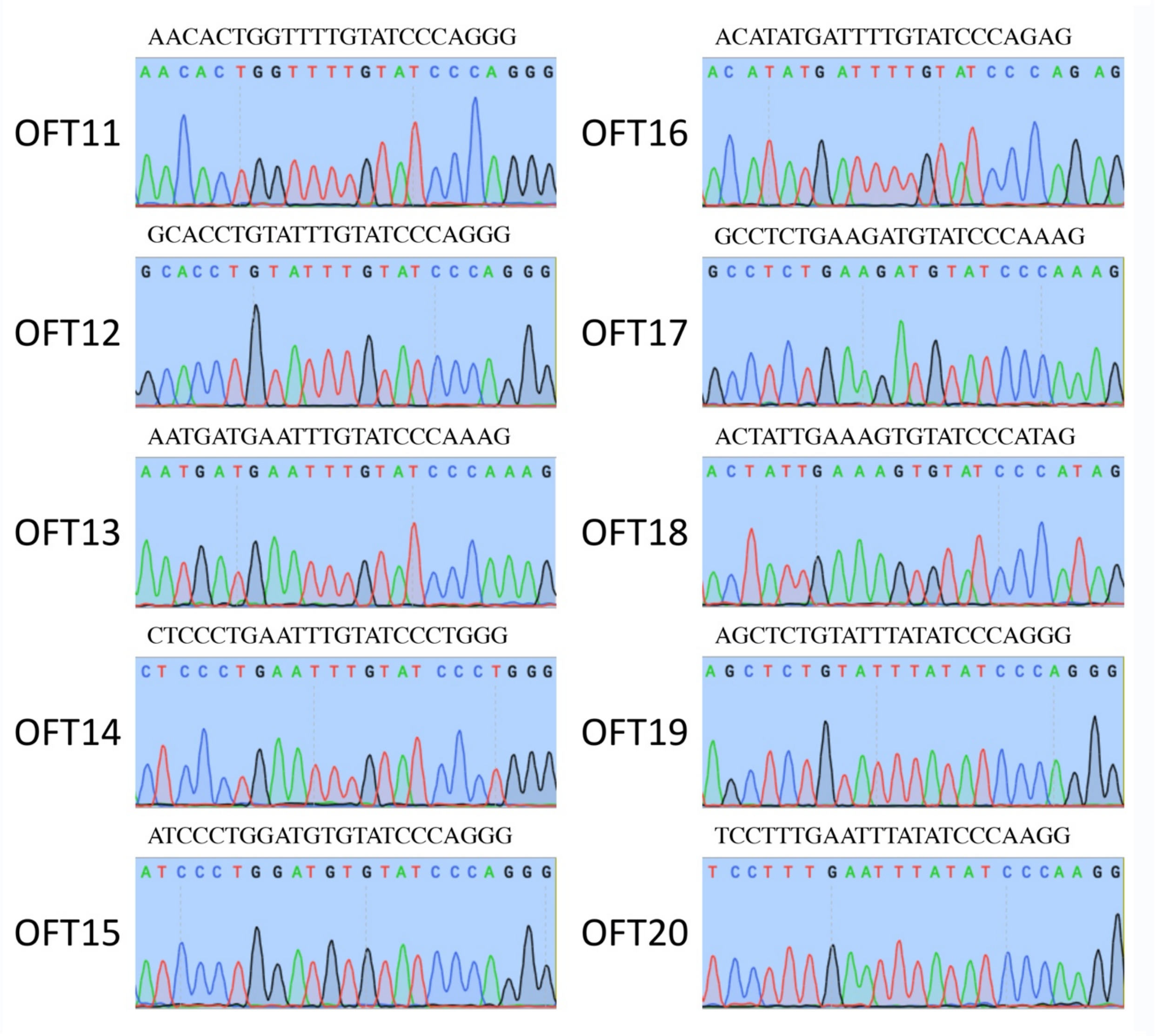

Supplemental Figure 1: IFN off-target analysis for IFNAR1 **(panel A)** and IFNAR2 **(panels B-E)**. Potential off-target sites were identified using the online Benchling software CRISPRtool (https://www.benchling.com/). Twenty potential off-target sites, ten per gene, were amplified using the primers listed in Supplemental Table 1 and the PCR products were directly sequenced on an ABI 3730 sequencer. The sequences from the sheep genome database are shown above the Sanger sequencing chromatograms.
